# Predictors of functional impairment in patients with bipolar disorder

**DOI:** 10.1192/j.eurpsy.2022.427

**Published:** 2022-09-01

**Authors:** C. Kartal, E.F. Aydın

**Affiliations:** 1 Sivas Sample Hospital, Psychiatry Department, Sivas, Turkey; 2 Atatürk University Faculty of Medicine, Psychiatry Department, Erzurum, Turkey

**Keywords:** attachment, affective temperament, functional impairment, bipolar disorder

## Abstract

**Introduction:**

Psychosocial functioning is an an important issue in the follow-up processes of patients with bipolar disorder. Potential predictors of functional impairment in bipolar disorder may give a chance to improve functioning in this group of patients.

**Objectives:**

We aimed to assess the differences between patients with bipolar disorder and healthy controls due to childhood traumas, attachment styles, dysfunctional attitudes, affective temperaments and to assess which of these factors may significantly predict the overall functional impairment in patients with bipolar disorder.

**Methods:**

63 remitted patients with bipolar disorder and 61 healthy controls were enrolled in the study. Asessment was conducted using a sociodemoghraphic and clinical questionnaire, Hamilton Depression Rating Scale 17-item version (HAM-D-17) and the Young Mania Rating Scale (YMRS), Childhood Trauma Questionnaire(CTQ-28), Relationship Scales Questionnaire (RSQ), Dysfunctional Attitudes Scale (DAS), Temperament Evaluation of Memphis, Pisa, Paris and San Diego (TEMPS-A) and Functioning Assesment Short Test (FAST).

**Results:**

In the patient group scores of childhood traumas, dysfunctional attitudes, cyclothymic, depressive and anxious temperaments, all domains of functional impairment scores except financial issues, and overall functional impairment scores were significantly higher than the control group. Besides this, secure attachment scores were significantly higher in the control group. In the regression analysis anxious temperament and subclinical depressive symptoms significantly positively predicted functional impairment and hyperthymic temperament significantly negatively predicted functional impairment in patients with bipolar disorder.

**Conclusions:**

In the assessment of functioning of patients with bipolar disorder subclinical depressive symptoms, hyperthymic and anxious affective temperament styles might be taken into consideration.

**Disclosure:**

No significant relationships.

